# Study on the mechanical behavior and rupture characteristics of coal after thermal damage

**DOI:** 10.1371/journal.pone.0315468

**Published:** 2024-12-16

**Authors:** Chuan Li, Yiping Zhang, Haijiao Bu, Hong Lan, Xianwei Heng

**Affiliations:** 1 College of Mining, Guizhou University, Guiyang, PR China; 2 Guizhou Vocational College of Culture and Tourism, Guiyang, PR China; 3 Guizhou Lindong Coal Industry Development Co., Ltd. Longfeng Coal Mine, Jinsha, Guizhou, PR China; Shenyang Jianzhu University, CHINA

## Abstract

High temperature is a critical safety concern that poses challenges to the safe and efficient operation of coal mines. To explore the mechanical behavior and fracture mechanisms of coal exposed to high temperatures, coal samples were subjected to various thermal treatments. After cooling, uniaxial compression tests were performed using an electronic universal testing machine to assess their macroscopic properties. A discrete element numerical model, reflecting the same mineral composition, was then developed to investigate the microscopic fracture behavior of thermally treated coal under uniaxial compression. The results indicate that at high temperatures, thermal motion becomes more pronounced, leading to increased particle displacement and a transition in coal failure from brittle to ductile behavior. High temperatures intensify thermal damage, generating numerous thermal cracks, which prolong the crack closure phase and delay the onset of the elastic deformation stage. Furthermore, the formation and propagation of these thermal cracks significantly influence macroscopic mechanical properties. Peak stress and elastic modulus decrease with rising temperature, with the most pronounced reductions occurring between 200°C and 300°C, where the thermal damage factor peaks at 0.72. As the temperature increases, the proportion of tensile cracks decreases, while shear cracks become more prevalent. Under heat treatment, tensile failure dominates, whereas shear failure is predominant during uniaxial compression. These findings provide valuable insights for improving safety protocols in high-temperature coal mining environments.

## 1. Introduction

Coal is a vital non-renewable energy resource, supporting the advancement of human society. However, as cumulative consumption rises annually, shallow coal reserves are becoming increasingly depleted, prompting a shift toward deeper mining operations [1–3]. This transition introduces new environmental conditions for coal seam storage [4]. Once mined, coal is exposed to air, causing a continuous temperature increase that accelerates its progression to the self-heating stage, bypassing prolonged low-temperature oxidation phases [5–7]. Furthermore, activities such as heat injection, coalbed methane extraction, and underground coal gasification subject coal to high-temperature environments [8, 9]. Exposure to high temperatures induces thermal damage in coal, weakening the bonding between particles, affecting its mechanical properties, and potentially triggering spontaneous combustion. These processes compromise resource utilization and contribute to significant coal wastage [10–13]. Thus, investigating the damage mechanisms of coal under high-temperature conditions is essential for understanding its stability and mitigating associated risks.

Coal is a natural aggregate composed of various minerals [14]. When subjected to high temperatures, differences in the thermodynamic properties of these mineral components cause thermal expansion, the initiation of new microcracks within the coal structure, and alterations in its strength [15–17]. This phenomenon has prompted extensive research by numerous experts. Yang et al. [18] employed CT scanning and electron microscopy to quantitatively investigate crack evolution in coal during heating. Their study combined macroscopic and microscopic analyses with uniaxial compression tests to examine the impact of thermal damage on mechanical properties and the development of pores and fissures. Zhao et al. [19] used nanoindentation techniques to study the micromechanical properties of coals with varying metamorphic grades, discovering a linear relationship between hardness, fracture toughness, and elastic modulus. They observed that higher mechanical strength correlated with reduced fracture propagation and compression deformation. Zheng et al. [20] examined the mechanical behavior of long-flame and anthracite coals at different drying temperatures, revealing a significant reduction in peak stress as temperature increased, along with a rise in the fractal dimension of rock fragmentation. Jiang et al. [21] analyzed the effects of temperature and particle size on the rapid pyrolysis of coal using a thermogravimetric analyzer and a downward bed reactor. Hassani et al. [22] applied ultrasonic technology to quantify internal defects in coal, showing the influence of various factors such as coal rank, water saturation, porosity, and permeability on ultrasonic wave propagation, which reflects the mechanical properties of the material. While these studies, grounded in laboratory-based physical experiments, provide valuable insights into the role of temperature in coal damage mechanisms, they face inherent limitations. These include the difficulty of accurately capturing the complex evolution of fracture behavior under varying temperature conditions, challenges in replicating experimental results, and the inability to visually observe the microscopic fracture processes driven by interactions between mineral particles.

Numerical methods used to study the damage of rocks are primarily categorized into the finite element method (FEM) and the discrete element method (DEM) [23, 24]. The FEM is a powerful approach for modeling continuous large deformations in rocks, while the DEM offers distinct advantages in simulating the heterogeneity, fracture, and damage processes of rocks. DEM is particularly effective for addressing the complexities of non-uniform and discontinuous surfaces, enabling detailed visualization of fracture morphology and precise representation of crack propagation on a small scale. The core assumption of the DEM is that the model consists of numerous rigid particles. While this assumption has been debated, its feasibility has been validated through extensive applications, making it particularly suitable for analyzing rock fracture characteristics [25–27]. To further understand the influence of temperature on rock damage, Yuan et al. [28] developed a particle flow thermodynamic coupling model that aligns with the macroscopic and microscopic properties of shale. Their study examined the role of natural microcracks, confining pressure, and heating modes in the evolution of thermal cracks. Zhang et al. [29] applied a particle flow model to investigate the temperature-dependent structural and mechanical changes in coals with varying degrees of metamorphism. Their findings revealed that low-metamorphism coals exhibited the fastest thermal crack initiation and expansion rates during heating, whereas thermal cracks in moderately metamorphosed coals predominantly formed during the later heating stages. Additionally, at identical temperatures, highly metamorphosed coals displayed inferior mechanical properties compared to their middle- and low-metamorphism counterparts. Yu et al. [30] constructed a grain-based model using DEM software to analyze the impact of high strain rate loading on the failure of coal sandstones. Wang et al. [31] introduced a coupled thermal-force grain model capable of accurately simulating the thermal expansion, contraction, and macroscopic fracture patterns of granite. While these studies offer valuable insights into the mechanical damage and fracture mechanisms of rocks exposed to high temperatures, the microscopic mechanisms underlying thermal damage in coal remain insufficiently understood.

This study investigates the thermal damage behavior of coal by subjecting coal samples to heat treatment at various temperatures, followed by cooling to room temperature before conducting uniaxial compression tests. Additionally, a discrete element numerical simulation model, was developed to explore the microscopic fracture evolution of coal under high-temperature conditions. The findings aim to offer theoretical guidance for ensuring the safety of coal mining operations in high-temperature environments.

## 2. Uniaxial compression test of coal samples after thermal damage

### 2.1 Preparation of coal samples

The coal samples utilized in this study were sourced from the 9# coal seam of Longfeng Coal Mine in Jinsha County, Guizhou Province, China. The sample preparation adhered strictly to the standards set by the International Society for Rock Mechanics (ISRM) [32]. Each sample was made with dimensions of Φ50 mm×100 mm, and the ends and surfaces were carefully polished to maintain non-perpendicularity and non-parallelism within ±0.2 mm, ensuring precision and reliability in the test outcomes.

### 2.2 Test procedure

**Fig 1:**
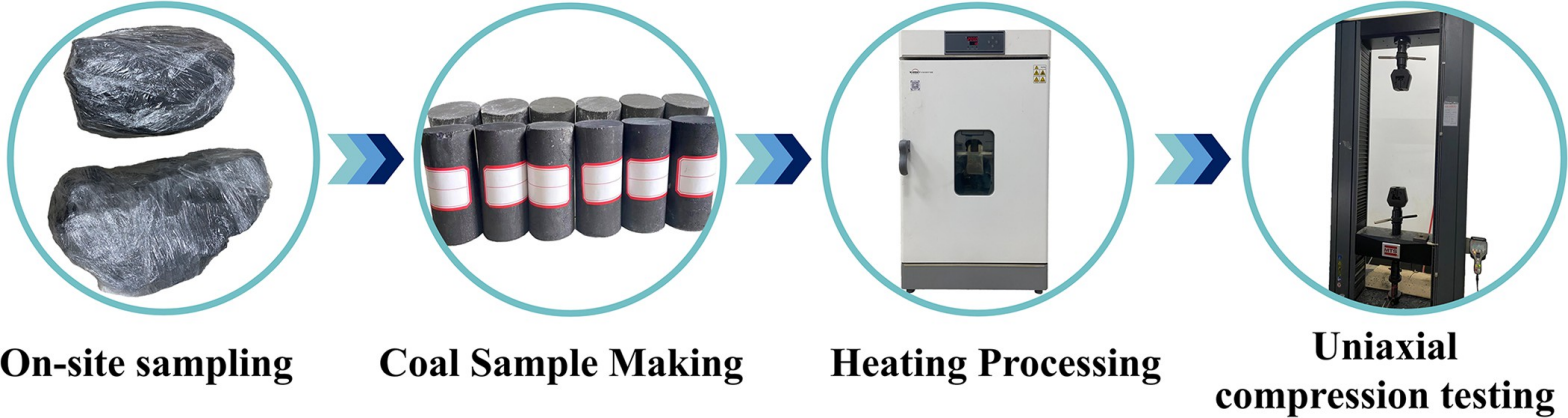
Flowchart of the test.

The coal samples underwent high-temperature treatment using an electric blast drying oven. To prevent excessive heating and the risk of spontaneous combustion when exposed to air, a flame-retardant coating, primarily made of magnesium hydroxide, was applied to the surface of each coal sample. This created a protective layer that acted as a barrier against flames. Six different temperature conditions were set for the test, including 25°C (ambient temperature), 100°C, 150°C, 200°C, 250°C, and 300°C, with each temperature condition being repeated three times. To avoid the generation of thermal shock, which could cause additional cracks in the coal samples, the heating rate was limited to a maximum of 10°C per minute. The samples were carefully placed in the heating chamber and gradually raised to the specified temperatures. Once each sample reached its target temperature, it was maintained at that temperature for 40 minutes to ensure uniform heating. After the 40-minute period, the samples were left to cool down naturally to room temperature through spontaneous combustion within the chamber. Following the heat treatment, uniaxial compression tests were performed using an electronic universal testing machine. The samples were subjected to a load rate of 50 N/S until they failed. The sequence of the testing process is illustrated in Fig 1.

### 2.3 Test results

**Fig 2:**
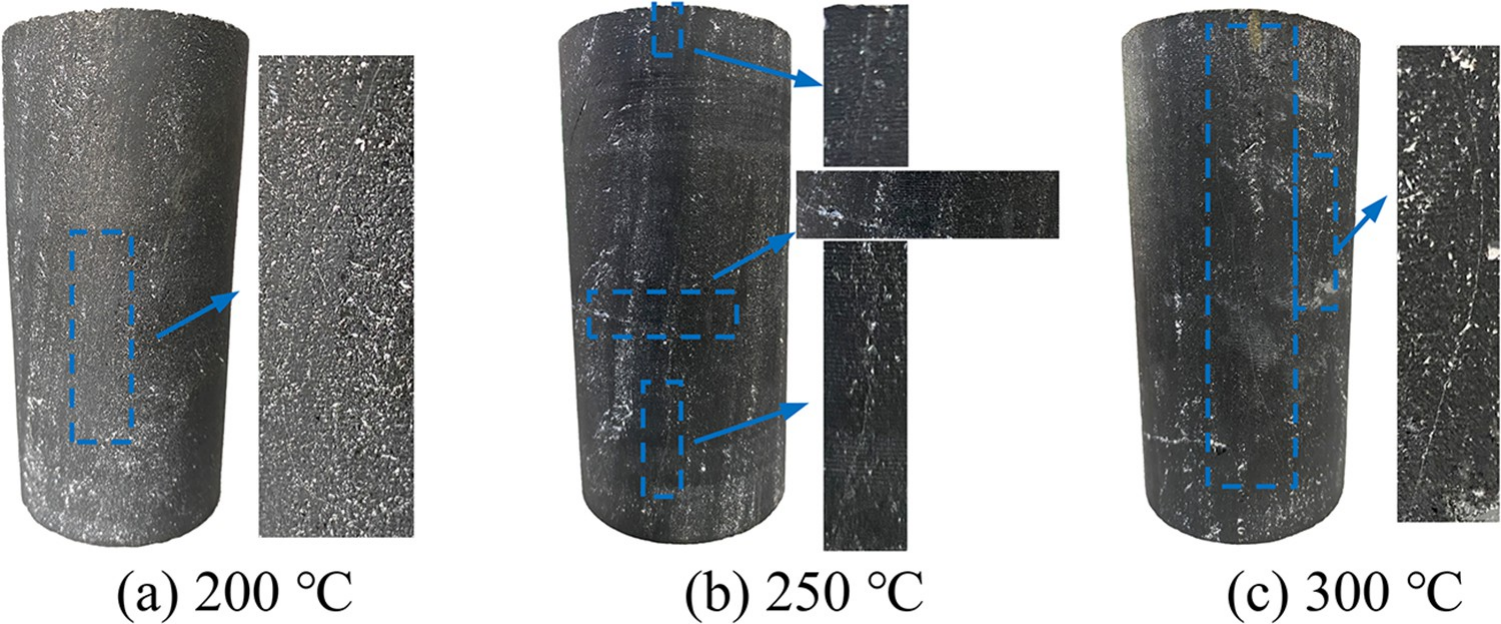
Microcracks on the surface of the coal sample.

During the heating process, strong-smelling gases such as sulfur dioxide and hydrogen sulfide are emitted. Between the temperature range of 150°C to 300°C, a distinct cracking sound could be heard, and as the temperature increased, the frequency of the sounds became more pronounced. After completing the thermal treatment, it was observed that coal samples exposed to temperatures between 200°C and 300°C developed thermal cracks on their surfaces (Fig 2). The quantity of cracks grew, and their length also expanded as the temperature rose, indicating that higher temperatures intensified the thermal damage to the coal samples.

The axial stress-strain curve of coal samples undergoes four distinct stages during testing [33]. The first stage is the crack closure stage, where the coal samples are compressed, and the primary pores or microcracks progressively close, leading to early nonlinear deformation. In this stage, the curve exhibits an upward bend, with the slope gradually increasing. The second stage is the elastic stage, where the stress-strain curve becomes nearly linear, and the slope of the line corresponds to the elastic modulus. The third stage is the stable crack development stage, where internal microcracks begin to form, but these cracks remain isolated from each other. The fourth stage is the unstable fracture development stage, characterized by a significant increase in the number of internal microcracks. These microcracks start to connect, gradually forming a macroscopic fracture surface, which ultimately leads to the complete failure of the sample.

**Fig 3:**
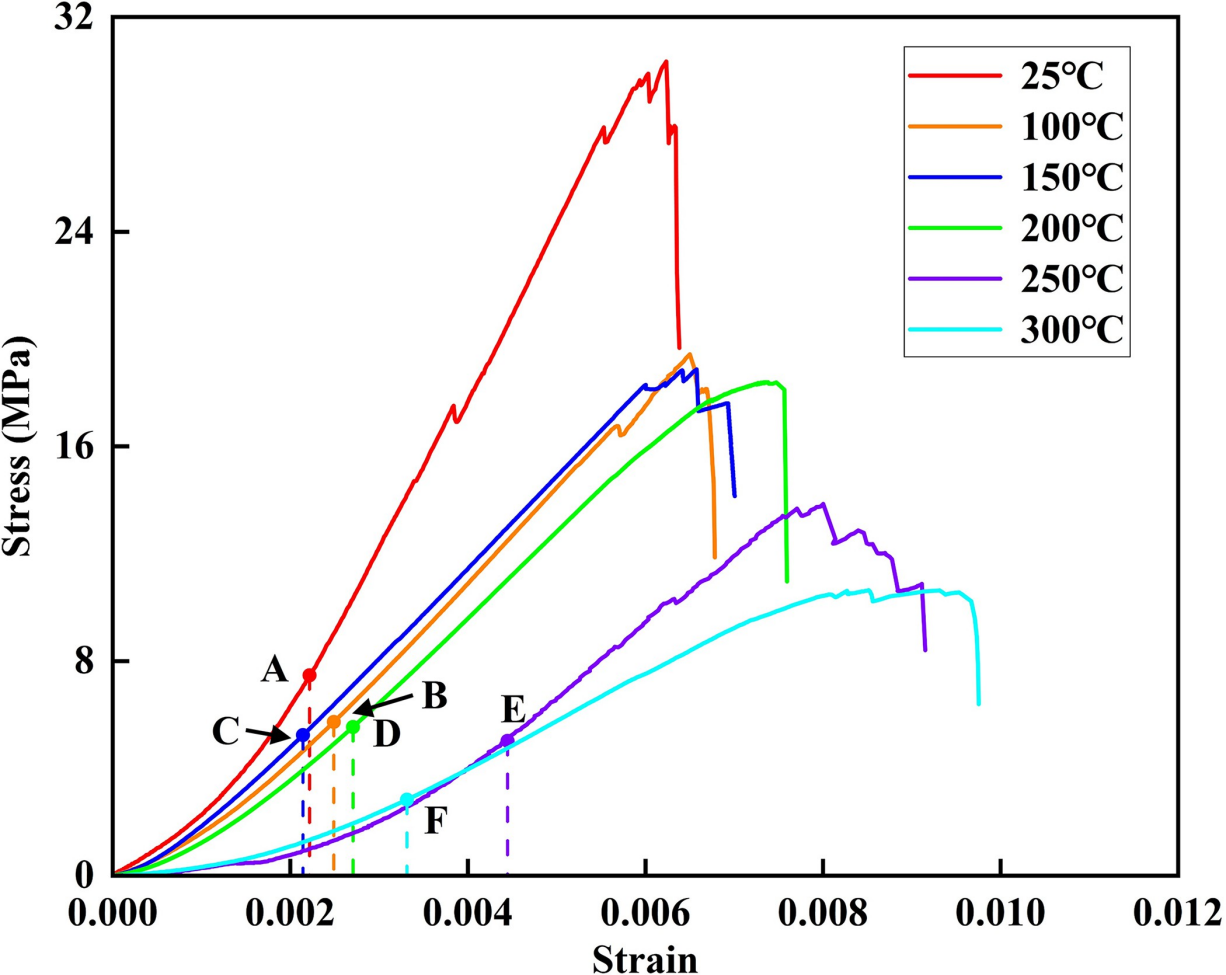
Stress-strain curves. OA, OB, OC, OD, OE, and OF represent the crack closure stage of the stress-strain curves at 25°C, 100°C, 150°C 200°C, 250°C and 300°C, respectively.

The stress-strain curves of coal samples treated at different temperatures are presented in Fig 3. It is observed that between 25°C and 200°C, the coal samples exhibit pronounced brittleness, characterized by a sharp decrease in stress after reaching the peak value, with only minimal changes in strain. However, at temperatures ranging from 250°C to 300°C, the mechanical behavior of the coal samples shifts from brittle to ductile. In this temperature range, after the peak stress is reached, the stress decreases gradually as strain increases. From a thermodynamic perspective, the thermal motion of particles becomes more pronounced with higher temperatures, weakening the bonds between the particles. This makes the particles more prone to displacement, resulting in reduced brittleness and increased ductility of the coal samples. Temperature changes also affect the crack closure stage of the coal. The crack closure duration follows this order: 250°C > 300°C > 200°C > 100°C > 25°C > 150°C. The elongation of the crack closure stage causes a rightward shift in the elastic stage. Except for the 150°C treatment, the elastic stage shifts rightward, with the most significant shift occurring at 250°C. The extension of the crack closure stage with increasing temperature suggests intensified thermal damage, leading to the formation of numerous microcracks within the coal.

## 3. Numerical model construction

### 3.1 Heat conduction theory

**Fig 4:**
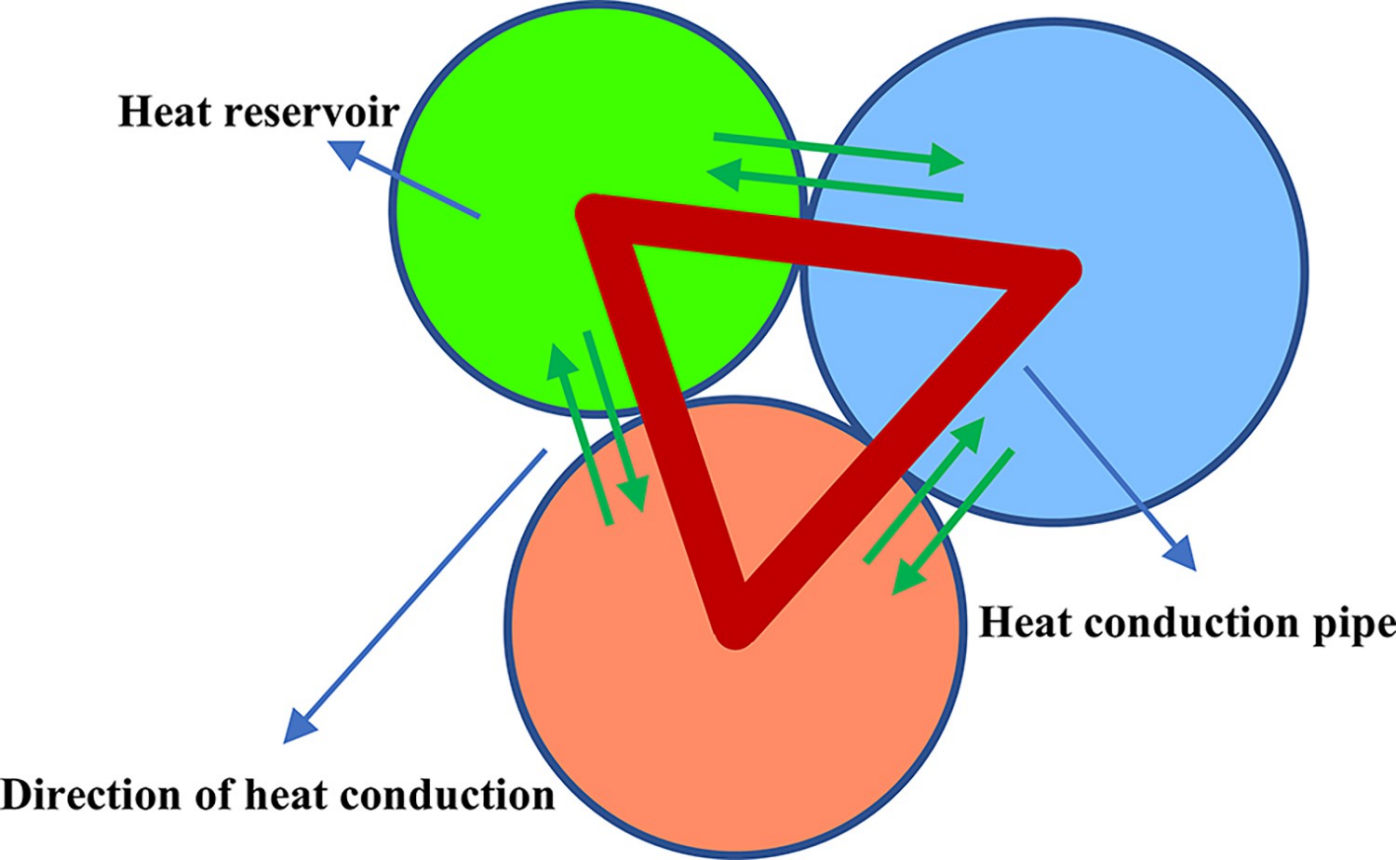
Schematic diagram of particle heat transfer flow conduction. (Circles denote heat reservoirs; red lines denote heat conduction pipes; orange circles denote quartz; bright green circles denote calcite; cornflower blue circles denote kaolinite).

In the Particle Flow Code (PFC), the rock is represented as a network of heat sources, which include heat reservoirs and heat conduction pathways. Each particle in the model corresponds to a heat reservoir, and the heat flux transfers between these reservoirs through the heat conduction pathways, as illustrated in Fig 4 [34, 35].

Assuming that the temperature change has a negligible effect on the strain, the continuous medium heat conduction equation for *n* heat conduction pipes can be expressed in the form below [36]:

$$-\sum_{1}^{n}Q+Q_{v}=mC\frac{\partial T}{\partial t}$$ 1

where *Q* represents the heat flow in the heat conduction pipe, *Q*_*v*_ denotes the heat flow intensity, *t* is the heating time, *m* signifies the reservoir mass, *T* is the temperature, and *C* is the specific heat capacity.

The heat flow *Q* can be calculated using the following expression:

$$Q=-\frac{T_{i}-T_{j}}{\eta L}$$ 2

the temperatures of the particles at the two ends of the pipe are denoted by *T*_*i*_ and *T*_*j*_, respectively, the thermal resistance per unit length is denoted by *η*, and the length of the heat conduction pipe is denoted by *L*.

The thermal resistance *η* in Eq. (2) can be expressed in terms of the thermal conductivity *K* in the following equation:

$$\eta =\frac{1}{2K}\left(\frac{1-\phi }{\sum N_{k}V_{k}}\right)\sum N_{p}l_{p}$$ 3

where *ϕ* represents the porosity, *V*_*k*_ is the volume of particles, *l*_*p*_ is the pipe length, and *N*_*k*_ and *N*_*p*_ are the number of particles and pipes, respectively.

The mechanical effect is assumed to occur instantaneously by thermal conduction. The thermal strain due to temperature change can be calculated by calculating the amount of change in the particle radius, the change in the particle radius can be expressed as [37]:

ΔR=αRΔT 4

where Δ*R* represents amount of change in radius, *α* denotes the thermal expansion coefficient of the particles, Δ*T* is the amount of change in temperature.

To model the impact of thermal expansion in rocks caused by temperature variations, it is assumed that temperature changes affect only the normal contact force, which is perpendicular to the contact surfaces during the bonding process. When a parallel bond is attached to a heat transfer pipe, the material experiences uniform expansion in all directions, changing the bond length *L*. Based on these changes, the component of the bonding force along the normal direction (Δ*F*_*n*_) can be expressed as:

$$\Delta F_{n}=-k_{n}\left(\alpha L\Delta T\right)$$ 5

where *k*_*n*_ represents the normal stiffness of the parallel bond.

### 3.2 Model validation

**Fig 5:**
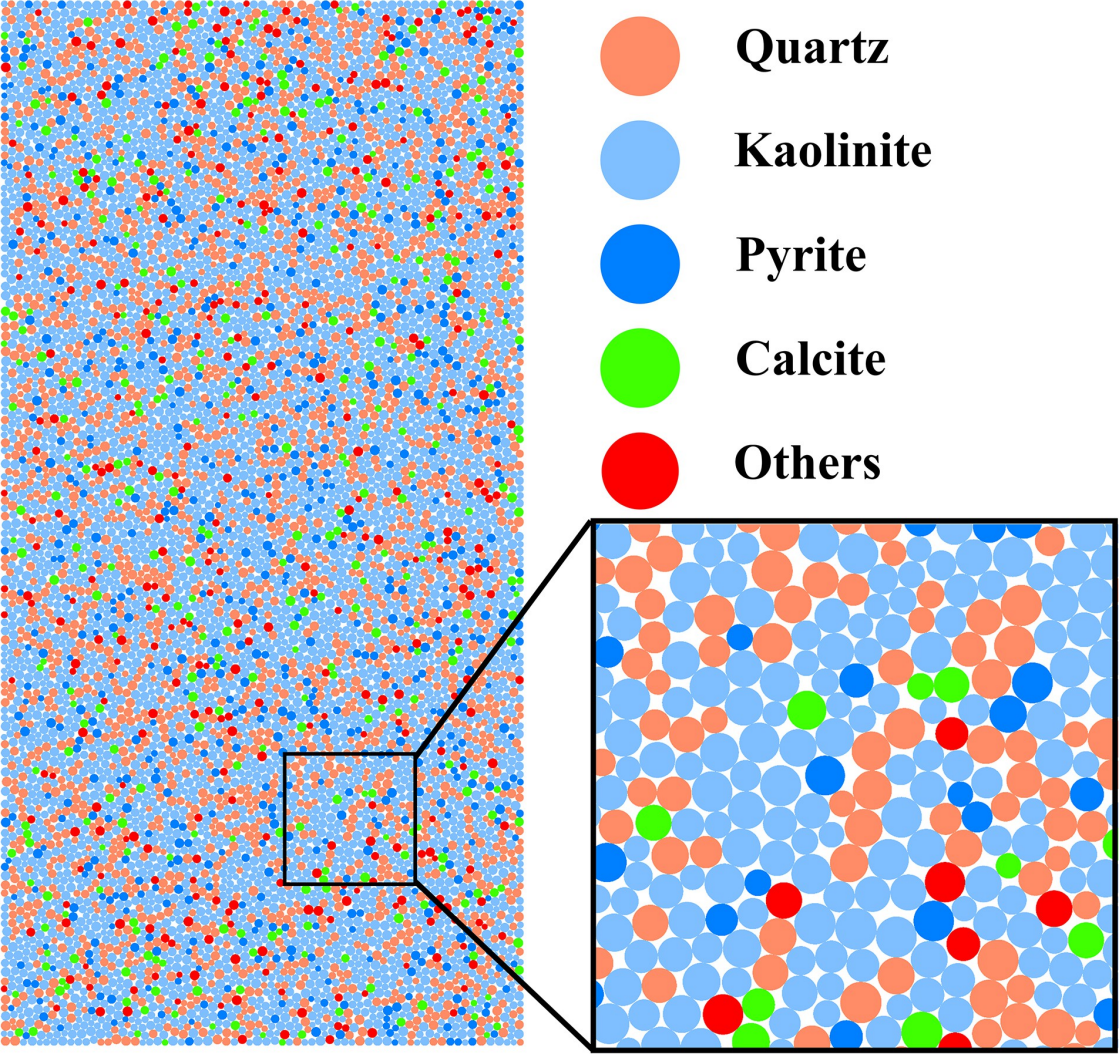
Discrete element characterization model. (Orange circles denote quartz; cornflower blue circles denote kaolinite; blue circles denote pyrite; bright green circles denote calcite; red circles denote other minerals).

A review of relevant literature reveals that the primary mineral components of the coal samples include quartz, kaolinite, calcite, and pyrite [38]. The thermal expansion coefficients and mineral contents of these minerals are presented in Table 1 [39, 40], and the specific heat capacity of the mineral fractions was set to 875 J/kg·°C. A numerical model was developed using cellular automata based on the proportions of the minerals [41], as depicted in Fig 5. The model was specified as a rectangle measuring 50 mm in width and 100 mm in length, containing particles with a minimum radius of 0.3 mm, and the ratio of the maximum diameter to this minimum radius was set to 1.66, reflecting the variation in particle size. The parallel bond contact model was chosen to replace the complex empirical behavior of the material with a simplified particle contact logic. This model facilitates the effective transfer of force and moments between particles, making it capable of simulating the mechanical response characteristics of rocks, thus offering an efficient and practical approach for rock mechanics research [42, 43]. In the study, the parallel bond mechanism was used to model the interactions between particles, with a total of 8801 particles and 17,397 parallel bonds generated in the model.

**Table 1:** Coefficients of thermal expansion of the mineral components and its contents.

Minerals	Coefficient of thermal expansion /(°C)^-1^	Composition ratio /%
Quartz	24.3×10^−6^	28
Kaolinite	6.16×10^−6^	56
Pyrite	14.0×10^−6^	8
Calcite	25.7×10^−6^	4
Others	19.6×10^−6^	4

To carry out the heat treatment simulations, the heating rate was set to 10°C per step, gradually raising the temperature to the target value, T. Concurrently, the thermodynamic calculation was activated to simulate the heating process. The model was considered to have reached a steady state when the ratio of the average force acting on all particles to the maximum unbalanced force fell below a threshold of 1.0 × 10^−5^. Upon completion of the heating phase, the model temperature was set to -T, and the same algorithm applied during heating was used to simulate the cooling process until equilibrium was restored. Following this, external loads were applied to both the top and bottom ends of the model, and loading continued until 60% of the peak stresses that the model could withstand were reached, at which point the loading was terminated.

**Fig 6:**
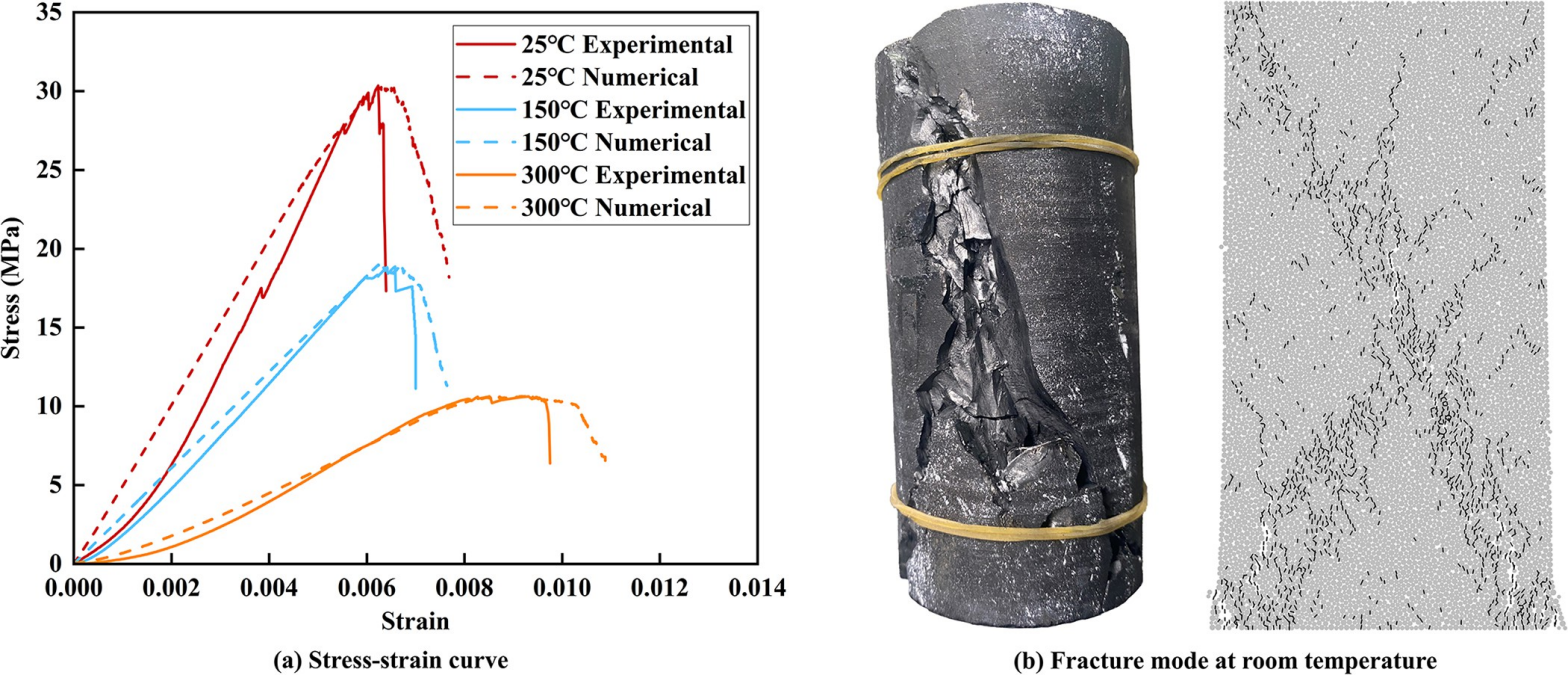
Comparison of uniaxial compression test and numerical simulation results.

After the initial numerical model was established, the peak stresses of coal samples at 25°C, 150°C, and 300°C under uniaxial testing were selected as the target values. The "trial and error method" was employed to iteratively compare the experimental results with the numerical simulation outcomes [44], allowing for the calibration of a set of feasible micro-mechanical parameters. Following multiple rounds of adjustments, the resulting stress-strain curve is shown in Fig 6(A), and the damage mode at room temperature is illustrated in Fig 6(B). A comparison of the numerical simulation results with the experimental data reveals a strong agreement, confirming the validity of the calibrated micro-mechanical parameters. The micro-parameters of the model are summarized in Table 2.

**Table 2:** Micro-parameters of the numerical model.

Micro-parameters	Value
Effective modulus of the particle, *E* (GPa)	5.52
Stiffness ratio of the particle, *k*_n_/*k*_s_	1.60
Friction coefficient, *μ*	0.60
Particle radius ratio, *R*_*max*_*/R*_*max*_	1.66
Tensile strength of the parallel bond, *σ*_c_ (MPa)	15.80
Effective modulus of the parallel bond, *E*_c_ (GPa)	5.52
Cohesion of the parallel bond, *c* (MPa)	15.80
Density, *ρ* (kg/m^3^)	1460.00
Porosity, *p*	0.10
Friction angle, *φ*	30.00

## 4. Results

### 4.1 Analysis of thermal cracks

**Fig 7:**
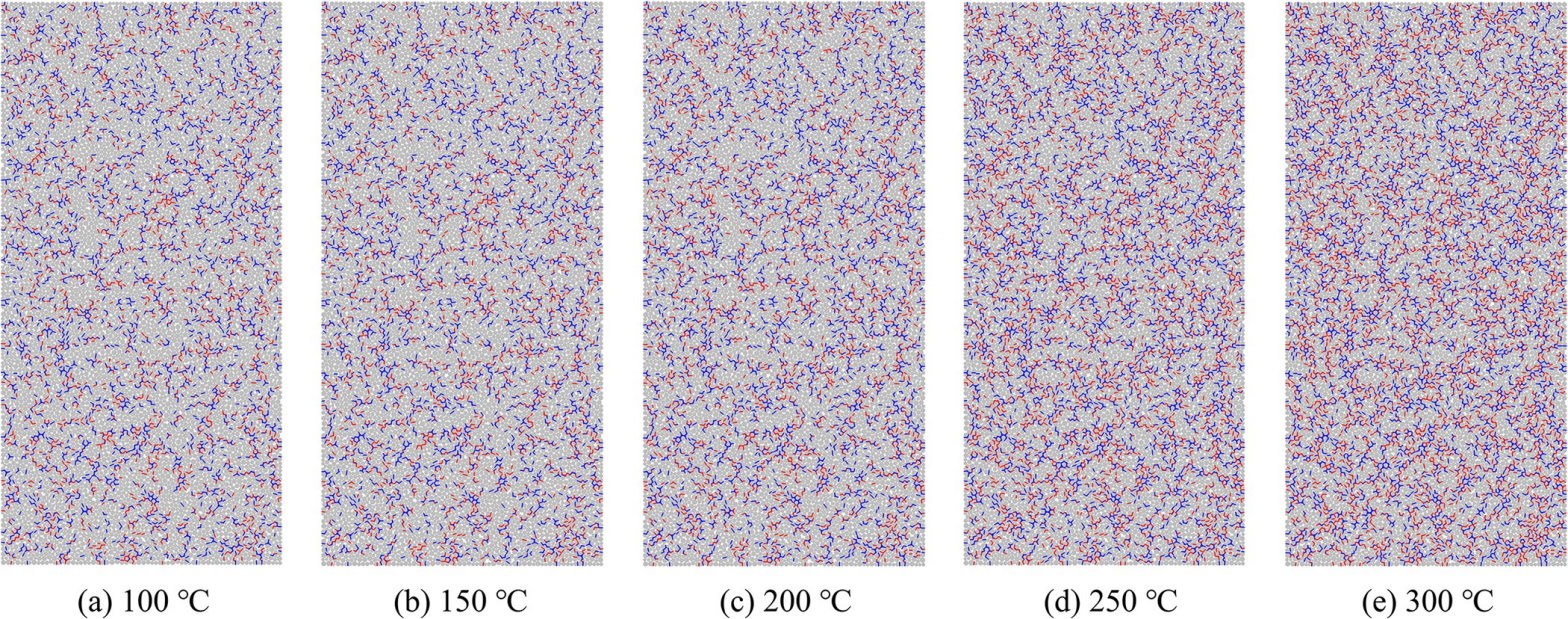
Distribution of thermal cracks.

**Fig 8:**
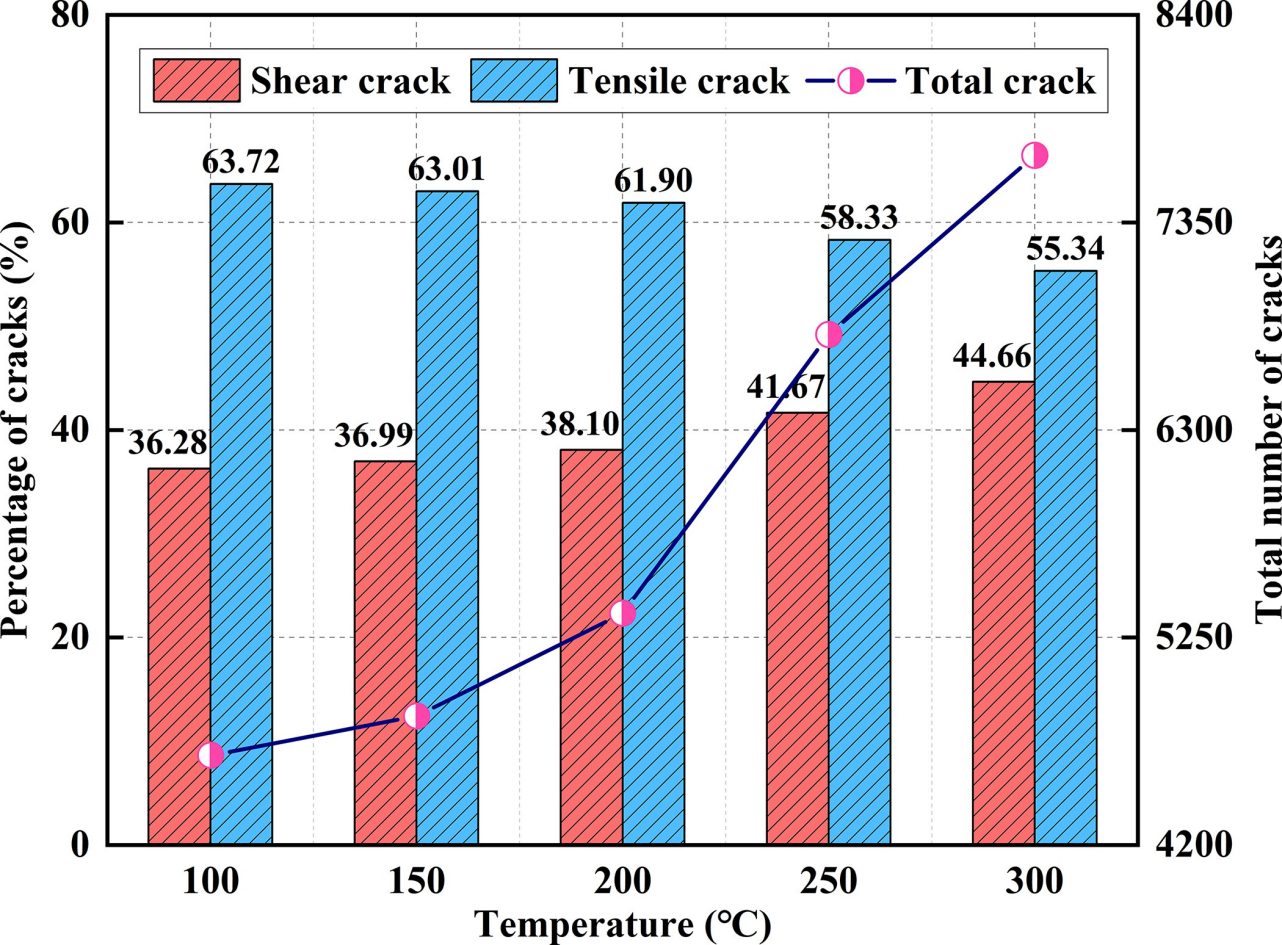
Number of thermal cracks in the model after the action of high temperature.

At high temperatures, the differential thermal expansion of the particles generates thermal stresses that reduce the bond strength between the particles. Once the applied stress exceeds the inter-particle bond strength, thermal cracks form between the particles, resulting in model damage. Fig 7 illustrates the distribution of thermal cracks at various temperatures. Fig 8 shows the progression of thermal cracks as the temperature increases, indicating that as the specimen experiences damage, microcracks form, and the total number of cracks increases, with the damage becoming irreversible. Between 100°C and 200°C, existing cracks expanded and new cracks formed, merging into a crack network with enhanced connectivity. Microcrack growth was halted at crack intersections, mineral grains, or pores, leading to a 15.42% increase in the total number of cracks. From 200°C to 300°C, the microcracks penetrated the mineral particles or pores, causing a significant increase in crack formation. The cracks became more densely distributed, with a 43.12% rise in the total number of thermal cracks. After heat treatment, tensile cracks predominated, and both tensile and shear cracks increased in number. However, the proportion of tensile cracks gradually decreased, while the percentage of shear cracks increased, narrowing the gap between the two types of cracks, from a difference of 27.44% at 100°C to 10.68% at 300°C. This change indicates that rising temperatures alter the nature of thermal crack formation.

### 4.2 Analysis of mechanical characteristics

**Fig 9:**
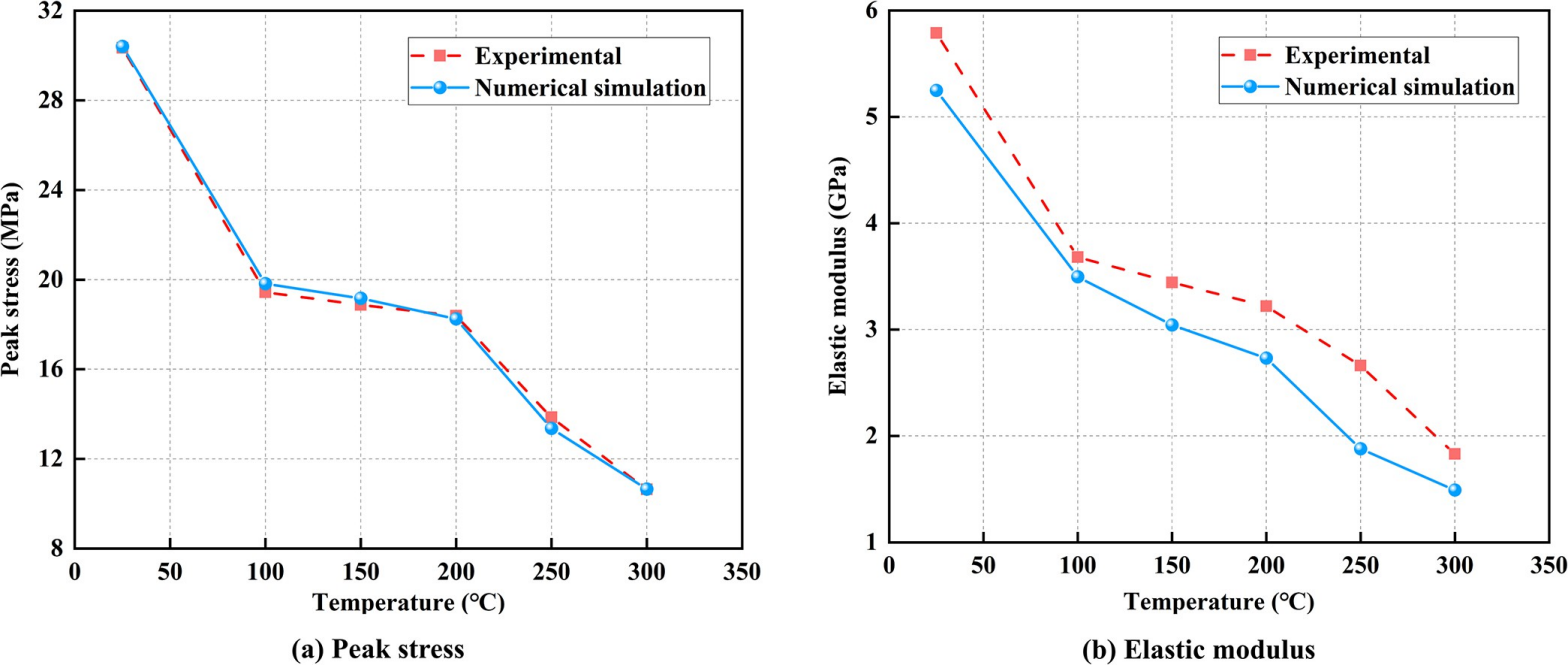
The Comparison of physical and mechanical parameters of coal samples obtained from numerical simulation and experiments.

Fig 9 indicates that both the elastic modulus and peak stress from the physical tests and numerical simulations follow a similar trend, decreasing as the temperature rises. The analysis primarily focuses on the indoor test results, dividing the temperature range into three stages: 25°C to 100°C, 100°C to 200°C, and 200°C to 300°C. Between 25°C and 100°C, the evaporation of water within the coal samples and the release of free gases adsorbed in the pores lead to the formation of many new cracks. This significantly reduces both the peak stress and elastic modulus, with decreases of 35.94% and 36.39%, respectively. From 100°C to 200°C, thermal decomposition of the coal occurs, during which the uneven expansion of minerals and gas release causes some cracks to close. However, the overall crack network continues to grow, reducing the structural integrity of the coal. As a result, the coal’s bearing capacity and deformation resistance decrease, with peak stress and elastic modulus dropping by 5.39% and 12.55%, respectively. From 200°C to 300°C, the internal structure of the coal undergoes extensive damage due to the crystalline transformation of minerals, thermal rupture, and oxidation of organic matter. Thermal interactions between particles cause the coal to fragment, cracks penetrate mineral particles or pores, and the number of cracks increases significantly. This results in the largest reductions in peak stress and elastic modulus, with decreases of 42.13% and 43.14%, respectively.

### 4.3 Microscopic displacement field and crack field analysis

**Fig 10:**
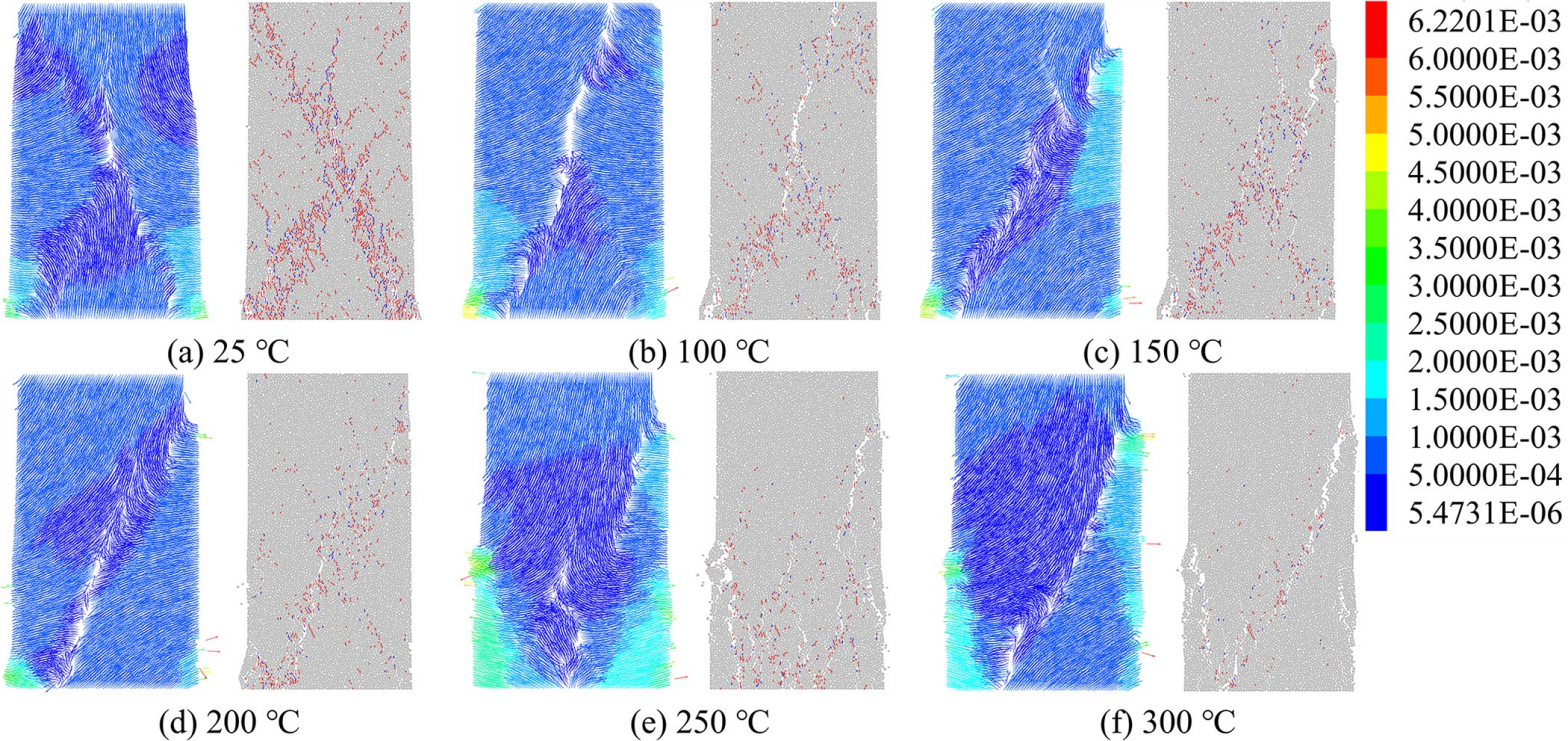
Crack field and displacement field of the model under uniaxial compression.

Fig 10 displays the particle displacement field and crack field of the model after uniaxial compression. The extension of microcracks influences the distribution of the displacement field, with areas of crack concentration primarily occurring where large displacement differences are observed. In the absence of lateral restraints, the edge particles exhibit more significant displacement than the particles in the center. This effect becomes particularly pronounced between 250°C and 300°C, where the regions with large displacement on either side of the model expand. At room temperature, the cracks form an overall "λ" shape. However, at 100°C, thermal stresses cause the upper crack to shift from the upper left to the center, resulting in a "Y"-shaped crack pattern. Between 150°C and 200°C, the crack on the upper side of the model moves to the upper right corner. At 250°C, the cracks concentrate mostly on the lower side of the model, causing it to fragment into several smaller pieces. By 300°C, the cracks at the upper right and lower left corners merge, resulting in better penetration, and the macroscopic damage pattern is characterized by shear failure.

**Fig 11:**
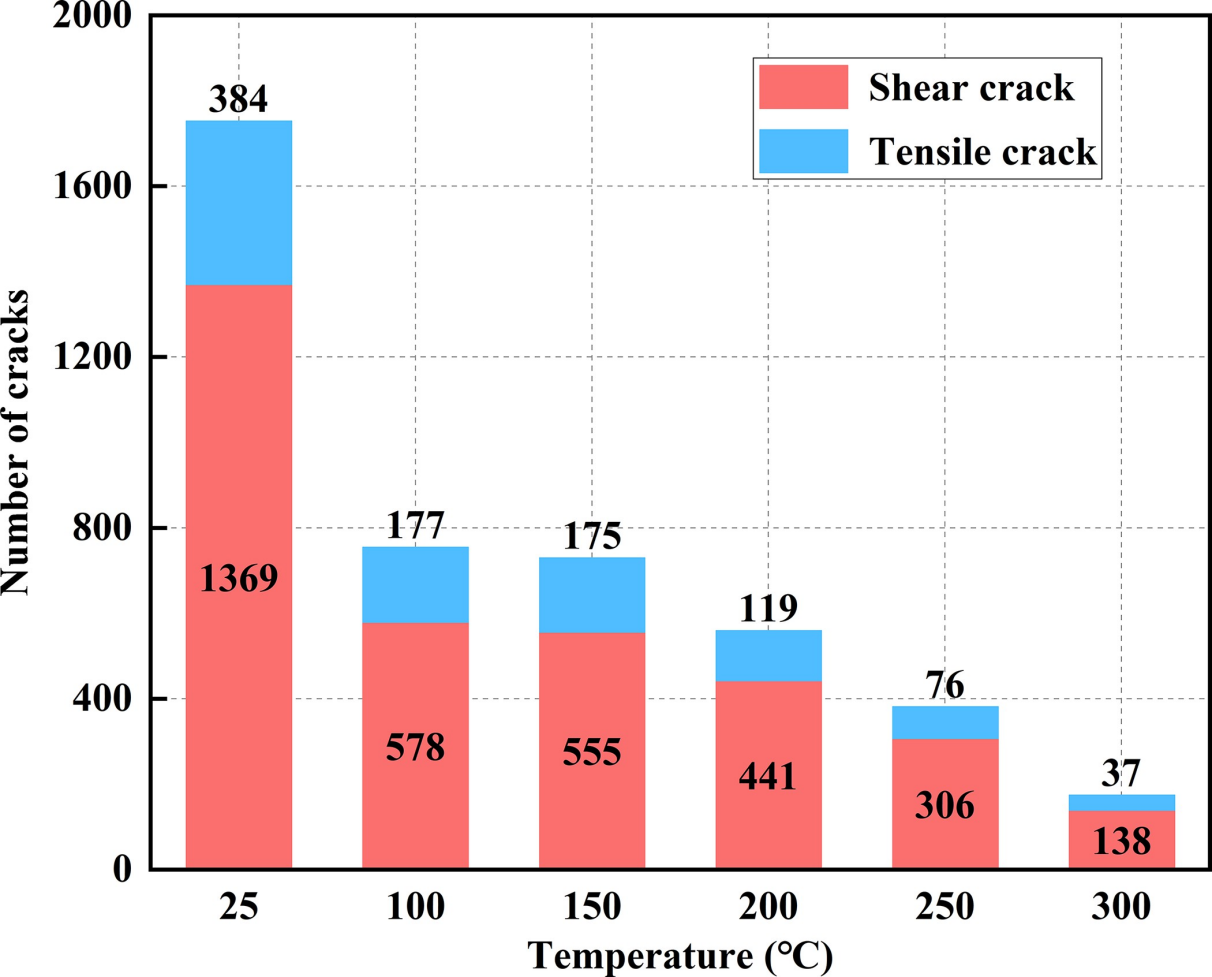
Number of cracks in models under uniaxial compression.

Fig 11 illustrates the change in the number of cracks in the models after uniaxial compression, with shear cracks being the dominant crack type, accounting for 76.03% to 80.10% of the total. The total number of cracks decreases as the temperature increases. Specifically, the number of cracks decreases by 56.93% at 100°C and by 90.02% at 300°C compared to the room temperature condition. This indicates that high temperatures have a significant impact on the thermal damage of the models, reducing the number of cracks produced during the uniaxial compression stage and making the models more susceptible to damage.

### 4.4 The fractal dimension of cracks

The distribution of cracks in coal subjected to high temperatures and uniaxial compression exhibits notable self-similarity. This characteristic makes it possible to use the fractal dimension as a quantitative metric to evaluate the extent of crack development in coal samples. Among the various techniques available for determining the fractal dimension, the box-counting method is commonly applied for analyzing crack patterns [45, 46]. This approach involves overlaying the crack pattern with square boxes of side length *r*_*k*_, counting the number of boxes *N*_*k*_ required to cover the cracks, and calculating the fractal dimension *D* as the box size approaches zero. The formula for determining *D* is as follows:

$$D=\underset{r_{k}\to 0}{\lim}\frac{\mathrm{lg}N_{k}\left(r_{k}\right)}{\mathrm{lg}r_{k}}$$ 6

**Fig 12:**
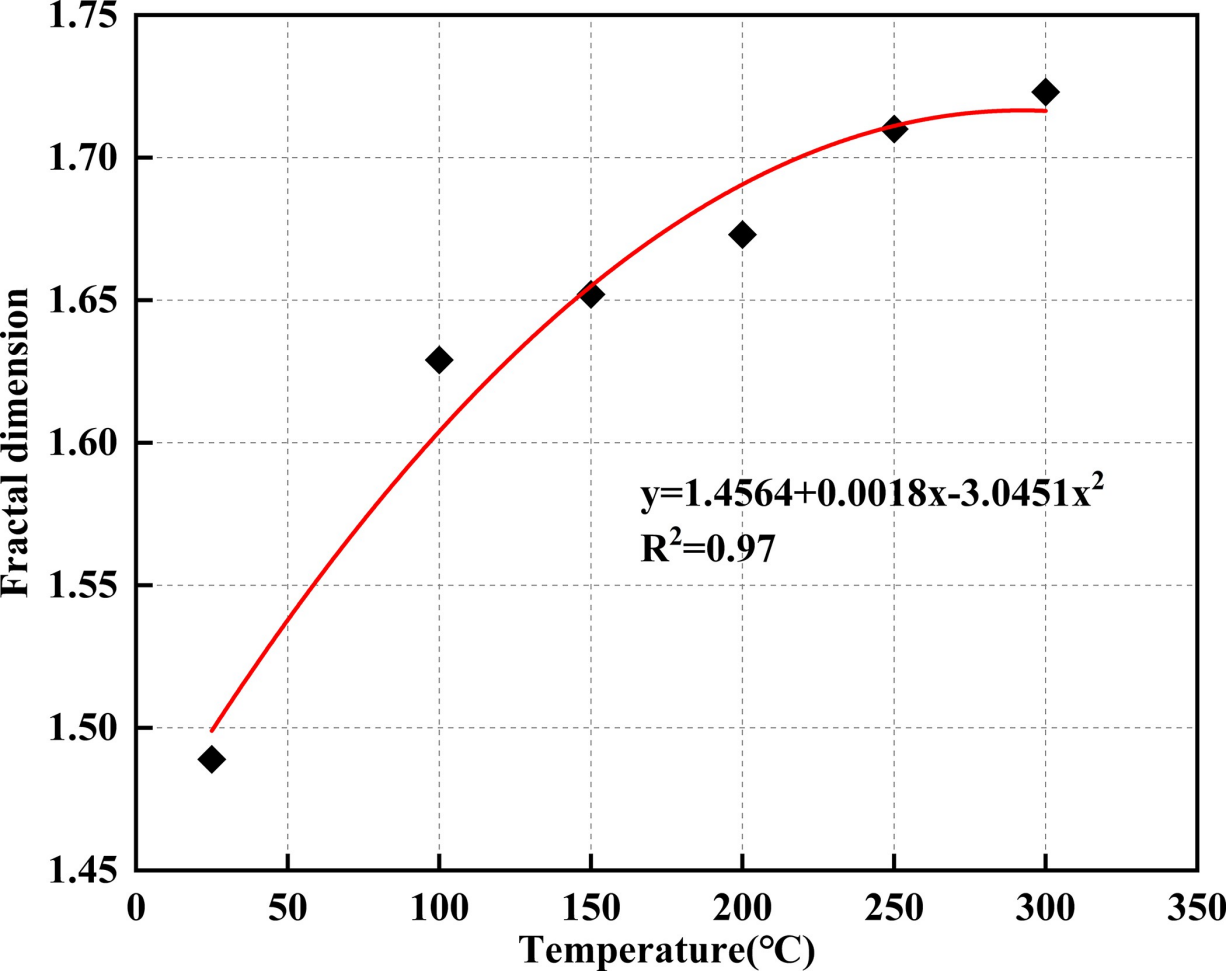
The fractal dimension of model cracks under different temperatures.

The crack images of the model, after undergoing uniaxial compression, were processed using MATLAB software. The images were first converted to grayscale and then binarized, reducing them to black and white for easier analysis. A series of square grids, each with the same side length r, were overlaid on the binary image to count the number of boxes that covered the cracks. This process was repeated with progressively smaller grid sizes, continuing until the side length of the boxes approached zero. The fractal dimension for the cracks at various temperatures was then determined by performing a linear fit between the box count and the side lengths of the boxes (Fig 12). The resulting fractal dimensions ranged from 1.489 to 1.723, exhibiting an exponential increase with rising temperature. This trend provides a quantitative representation of crack growth in the model. The most significant increase in fractal dimension occurred between 25°C and 100°C, while the rate of increase slowed when temperatures exceeded 100°C.

### 4.5 Relationship between thermal damage and temperature

The damage in coal induced by high temperatures is reflected in the formation of numerous microcracks within its structure. As the temperature rises, these microcracks expand and interconnect, leading to a progressive reduction in the elastic modulus. To quantitatively assess the extent of thermal damage at various temperatures, the elastic modulus is used to define the thermal damage factor *D*_*T*_. This factor serves as an indicator of the degree of thermal degradation experienced by coal under different thermal conditions [47, 48].

$$D_{T}=1-\frac{E_{T}}{E_{O}}$$ 7

where *E*_*T*_ and *E*_0_ denote the elastic modulus of coal samples at temperature *T* and at 25°C, respectively.

**Fig 13:**
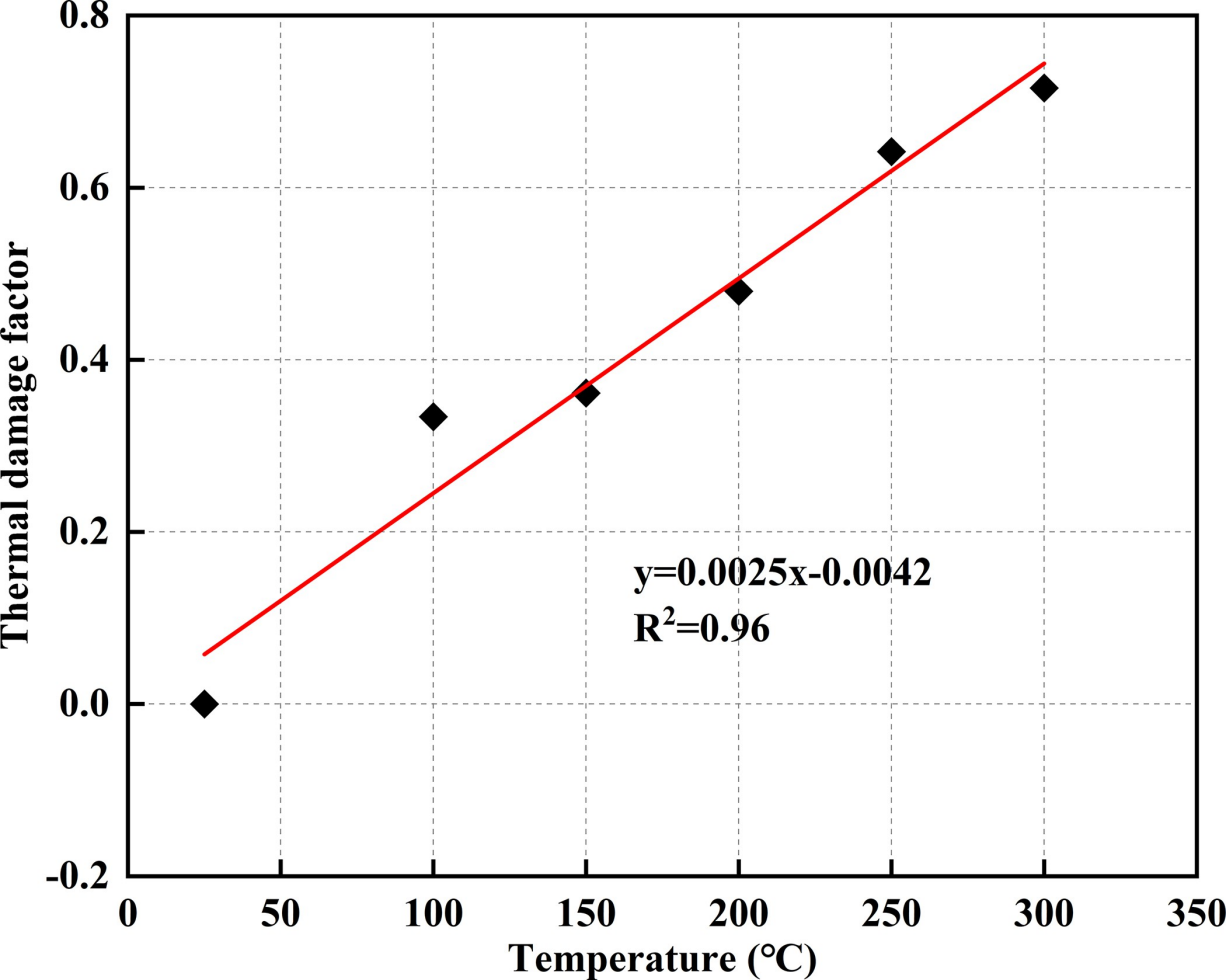
Relationship between thermal damage and temperature.

The correlation between thermal damage in coal samples and temperature is illustrated in Fig 13. The thermal damage factor demonstrates a strong linear relationship with temperature, progressively increasing as the temperature rises. At 300°C, the damage factor reaches its maximum value of 0.72, indicating the highest level of thermal degradation.

## 5. Discussion

Previous research has primarily focused on analyzing how temperature changes influence the modulus of elasticity, yet the relationship between variations in the modulus of elasticity and coal fracture behavior has received little attention. The modulus of elasticity represents a rock’s resistance to deformation, and in numerical simulations, it can be adjusted by modifying the model’s microscopic parameters. This approach effectively simulates changes in the mechanical properties of coal with varying elastic moduli after heat treatment. Furthermore, numerical simulations often repeat tests on the same coal sample, overlooking the influence of factors such as mineral composition, moisture content, and structural characteristics on the mechanical properties and fracture behavior of coal. This study assumes uniform mineral composition across all coal samples and excludes the effect of variability in mineral content on coal properties after heat treatment. The analysis will focus on the individual impacts of elastic modulus and mineral composition on coal fracture behavior, along with the engineering implications of thermally damaged coal.

### 5.1 Variation of crack number and stress in coal with the different modulus of elasticity

**Fig 14:**
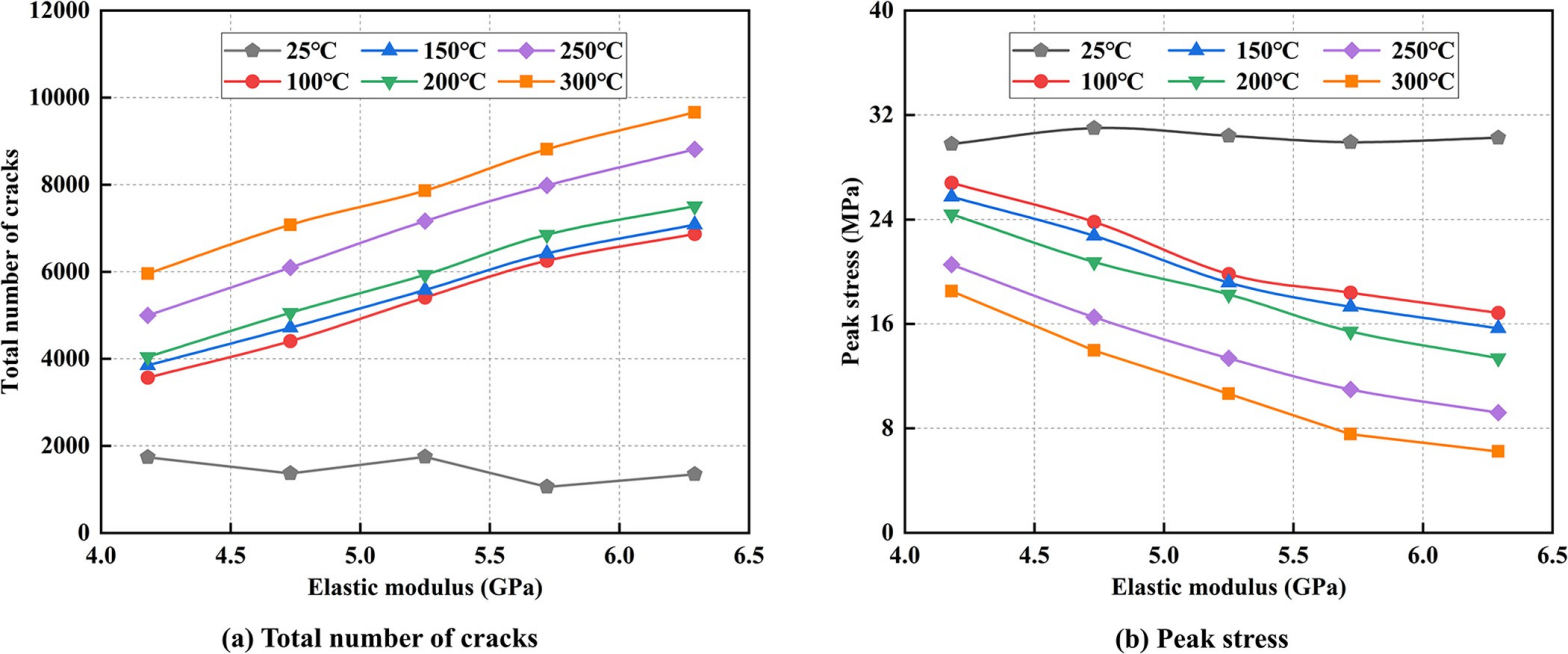
The relationship between temperature and crack number, peak stress for different modulus of elasticity.

The modulus of elasticity was assigned values of 4.18 GPa, 4.73 GPa, 5.25 GPa, 5.72 GPa, and 6.29 GPa. Fig 14 illustrates the relationship between temperature and peak stress, as well as the total number of cracks, corresponding to these varying elastic modulus values.

Fig 14(A) reveals that at room temperature, the total number of cracks tends to stabilize. Outside of room temperature, the total number of cracks exhibits a linear relationship with the elastic modulus at the same temperature, increasing as the elastic modulus rises. Additionally, the total crack count grows with rising temperature for a given elastic modulus, and the difference in crack numbers between room temperature and high-temperature conditions becomes more pronounced with higher elastic modulus values. Fig 14(B) shows that at room temperature, peak stress remains relatively constant despite increases in the elastic modulus. However, following heat treatment, peak stress progressively decreases as the elastic modulus increases. This suggests that while the elastic modulus has minimal impact on the model’s compressive strength at room temperature, it exerts a more significant influence after exposure to elevated temperatures.

### 5.2 Variation in the number of cracks and stresses in coal with different mineral contents

Variations in mineral composition and content can influence the mechanical properties and fracture behavior of coal. To investigate this, a control group was established with a higher proportion of quartz and kaolinite compared to the coal samples used in this study, while the content of other mineral components was relatively reduced. The model’s micro-parameters remained unchanged for consistency, and the mineral content details are provided in Table 3.

**Table 3:** Mineral component content.

Minerals	Quartz	Kaolinite	Pyrite	Calcite
Composition ratio /%	32	60	6	2

**Fig 15:**
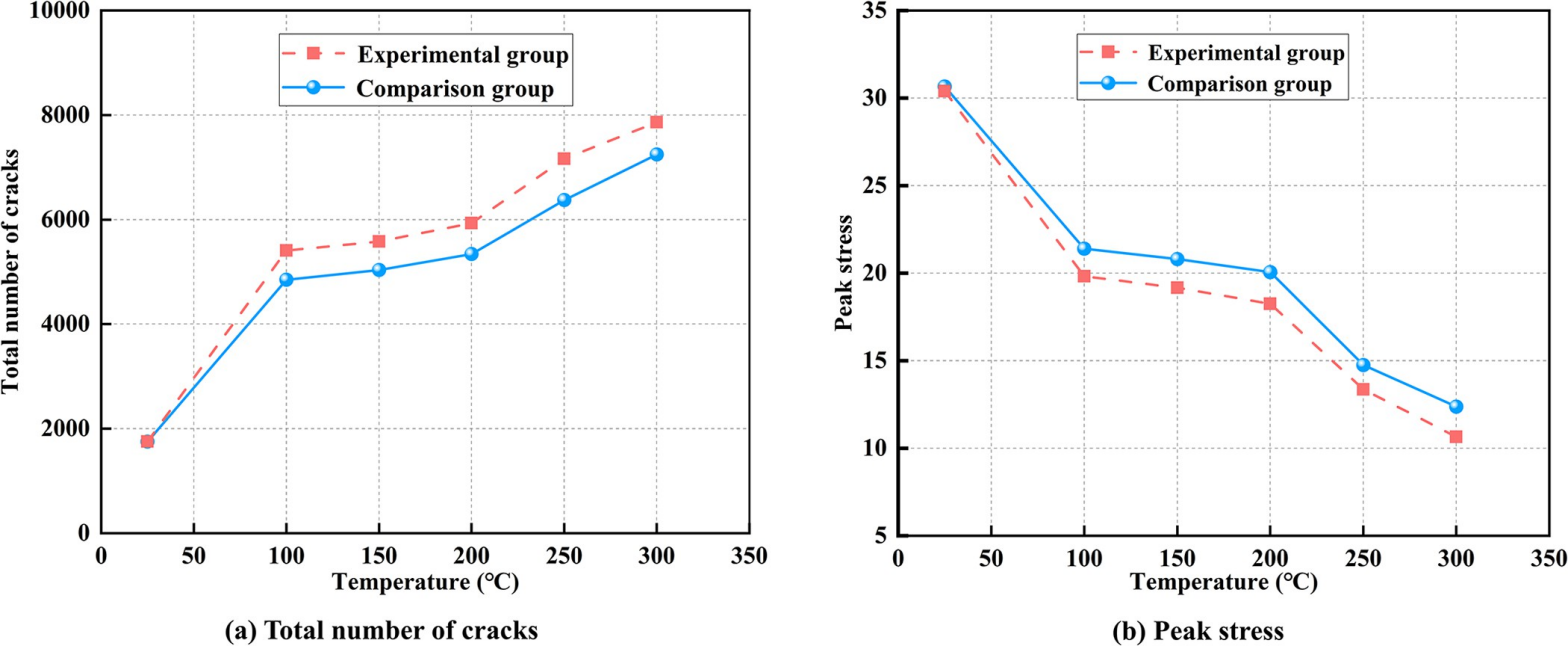
The relationship between temperature and crack number, peak stress for different mineral content.

Fig 15 shows that the control group model generates fewer cracks and exhibits higher peak stress within the temperature range of 25°C to 300°C compared to the coal samples analyzed in this study. This suggests that an increase in quartz and kaolinite content can suppress crack formation while enhancing the strength of the coal structure. However, the precise relationship between the mineral composition content, coal strength, and fracture behavior under high-temperature conditions remains unclear. Investigating these relationships will be the primary focus of future research.

### 5.3 Potential engineering applications of coal under thermal damage

As mining activities extend to greater depths, advanced processes such as coalbed methane injection for heat extraction and underground coal gasification have become increasingly prevalent. These processes lead to elevated temperatures within coal seams, making the study of temperature effects on coal rupture behavior and mechanical properties a crucial but challenging area of research, especially for practical engineering applications. Based on the findings of this study, the following suggestions and improvement measures are proposed for field engineering applications:

(I) Cooling Measures: Exposure to high temperatures causes coal to transition from brittle to ductile, resulting in reduced mechanical strength, which can facilitate coal extraction. However, high-temperature environments increase the risk of coal dust explosions during mining operations. To mitigate this risk, enhanced cooling of both the coal and equipment is essential to lower the temperature and prevent such explosions.

(II) Gas Management and Prevention: Elevated temperatures in coal seams lead to an increase in internal cracks and alterations in the pore structure, creating more pathways for coalbed methane (CBM) to migrate and diffuse. This improves CBM permeability and extraction efficiency but also raises the risk of gas outbursts in high-temperature conditions. To address this, it is crucial to intensify gas monitoring and implement effective gas extraction strategies to prevent gas outbursts.

(III) Monitoring Coal Temperature: Temperature-induced thermal stresses, combined with changes in pore pressure during the mining process, can cause stress redistribution within the coal seam. Such stress variations may compromise the coal seam’s strength, particularly when temperatures exceed 200°C, increasing the likelihood of instability and failure. Continuous temperature monitoring is necessary to identify and address these risks promptly.

By applying the methods outlined above, along with advanced geological modeling and simulation techniques, it is possible to more accurately predict the formation and growth of cracks within the coal body, as well as the associated changes in its strength. The development of complex crack networks not only enhances CBM permeability but also boosts extraction efficiency. However, the successful implementation of these strategies requires careful consideration of geological conditions, engineering capabilities, and economic factors to optimize the use of temperature in gas extraction. Future research will continue to investigate the feasibility and practical effectiveness of these approaches.

## 6. Conclusions

This study analyzed the macroscopic mechanical properties of coal subjected to high temperatures by exposing coal to various temperature levels and performing uniaxial compression tests. A discrete element numerical simulation model was developed, incorporating mechanical parameters and fracture modes, to investigate the thermal damage behavior of coal under elevated temperatures and the microscopic fracture characteristics under loading. The key findings of the study are summarized as follows:

(1) In the uniaxial compression tests of coal samples subjected to heat treatment, as the temperature increases, the damage behavior of coal shifts from brittleness to ductility. Additionally, higher temperatures intensify the thermal damage to the coal, increasing microcracks and extending the crack closure phase of the uniaxial compression test, while delaying the elastic stage.

(2) The results from the numerical simulation tests show that the crack fractal dimension increases exponentially with rising temperature. Thermal cracks induced by heat treatment are primarily tensile in nature, but as the temperature increases, the proportion of shear cracks rises, and the proportion of tensile cracks decreases. However, under uniaxial compression tests, shear cracks dominate, and the formation of cracks due to loading decreases as the number of thermal cracks increases.

(3) As the heat treatment temperature rises, the degree of thermal damage to the coal increases, reducing its strength and ability to resist elastic deformation. In the temperature range of 200°C to 300°C, the increase in the number of thermal cracks was the highest at 43.12%, and the largest reductions in the modulus of elasticity and peak stress were 43.14% and 42.13%, respectively. Thermal damage to the coal samples exhibited a linear relationship with temperature, with the thermal damage factor reaching a maximum value of 0.72 at 300°C.
